# Changes in the gut microbiome associated with infliximab in patients with bipolar disorder

**DOI:** 10.1002/brb3.2259

**Published:** 2021-06-21

**Authors:** Aadil Bharwani, Jake C. Szamosi, Valerie H. Taylor, Yena Lee, Asem Bala, Rodrigo Mansur, Mehala Subramaniapillai, Michael Surette, Roger S. McIntyre

**Affiliations:** ^1^ Michael G. DeGroote School of Medicine McMaster University Hamilton Ontario Canada; ^2^ Department of Medicine McMaster University Hamilton Ontario Canada; ^3^ Department of Psychiatry, Foothills Medical Centre University of Calgary Calgary Alberta Canada; ^4^ Mood Disorders Psychopharmacology Unit University Health Network Toronto Ontario Canada; ^5^ Institute of Medical Science University of Toronto Toronto Ontario Canada; ^6^ Department of Medicine, Farncombe Family Digestive Health Research Institute McMaster University Hamilton Ontario Canada; ^7^ Department of Psychiatry University of Toronto Toronto Ontario Canada

**Keywords:** 16S rRNA sequencing, bipolar disorder, gut microbiota, inflammation, infliximab

## Abstract

**Objectives:**

Available information exists supporting the gut–brain axis, but additional information is needed to explore how the gut microbiome changes when exposed to mood disorder treatments. We sought to explore the effects of a novel treatment for bipolar disorder (BD), infliximab, on the gut microbiome.

**Methods:**

Participants with a primary diagnosis of BD (*n* = 15) who participated in a 12‐week, randomized placebo‐controlled trial evaluating the efficacy of adjunctive infliximab in the treatment of BD were recruited and followed. Stool samples were collected prior to randomization and at 12 weeks. 16S rRNA sequencing was employed in order to analyze the gut microbial community profile.

**Results:**

A total of 17 participants were randomized to infliximab (*n* = 9; mean [SD] age, 47.6 [10.3] years; 8 female) or to placebo (n = 8; mean [SD] age, 45.9 [10.7] years; 7 female) but two participants from the infliximab group were lost to follow‐up post randomization. Across all time points, there were no differences in the diversity on either Shannon or Simpson's Diversity indices. Comparison of Aitchison distances revealed a lack of clustering of the microbiota by time point, but did reveal a small overall effect of treatment that was not significantly different at individual time points. There were also no effects of either time or treatment on differential abundance at either the amplicon sequence variant or genus level.

**Conclusions:**

These observations indicate that no community‐wide changes in the microbiota diversity and profile were detected after the use of infliximab treatment.

## Graphical abstract

We sought to explore the effects of a novel treatment for Bipolar Disorder (BD), Infliximab, on the gut microbiome. A total of 17 participants were randomized to infliximab or to placebo and At 12‐weeks, the microbiota diversity of infliximab‐treated patients was not significantly different than that of placebo‐treated controls.

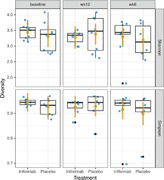

## 1 INTRODUCTION

There is a replicated body of literature linking bipolar disorder (BD) to alterations of the systemic immune system including low‐grade chronic inflammation and T‐cell activation (Anderson & Maes, 2015). There is also a compelling body of work linking the gut and the central nervous system (CNS) in what is now known as the microbiota–gut–brain axis, and immune changes have been hypothesized as one of the pathways mediating this interaction (Cryan & Dinan, 2012).

Several lines of compelling animal research supports this association: germ‐free (GF) mice exhibit altered expression of mRNA levels of brain‐derived neurotrophic factor (BDNF) and the 5HT1A receptor in the dentate gyrus, and the NR2B N‐methyl‐d‐aspartate (NMDA) receptor subunit in the amygdala (Neufeld et al., 2011), while administration of a specific bacterial strain to conventional mice for 28 days reduced anxiety‐like behavior and produced regional changes in brain gamma‐aminobutyric acid (GABA) receptor subtypes—effects that were abrogated by prior vagotomy (Bravo et al., 2011). The absence or modification of the microbiota in mice also influences the hypothalamic–pituitary–adrenal axis stress response and both anxiety‐ and depression‐like behaviors (Sudo et al., 2004). Furthermore, severe depressive illness is accompanied by biomarker evidence of a chronic low grade inflammatory state (Raison et al., 2010), and while efferent vagal stimulation downregulates inflammation through the cholinergic anti‐inflammatory pathway (Koopman et al., 2016; Olofsson et al., 2012), ingestion of specific bacteria selectively stimulates the T‐cell‐dependent anti‐inflammatory regulatory system (Neufeld et al., 2011). The potential role of the gut in the etiology and possible treatment of neurological and psychiatric diseases was also recently highlighted in studies showing the presence of Lewy bodies in the enteric nervous system in alpha synuclein knock‐in transgenic model and early‐stage Parkinson's disease, preceding central nervous system manifestations (Rietdijk et al., 2017; Sampson et al., 2016). These preclinical results are supported by studies on individuals with major depressive disorder (MDD), where recent work has identified a significant correlation between the gut microbiota and depression in 37 patients with depression and 18 nondepressed patients (Naseribafrouei et al., 2014). A second study also found a significant association between depression and the gut microbiome in 46 patients with depression (29 active‐MDD and 17 responded‐MDD) and 30 healthy controls, as well as increased bacterial diversity in the active depression group but not in the remitted depressed group (Jiang et al., 2015). Differences have also been noted in individuals with BD, and a recent study that compared 23 individuals with BD to a group of 23 health controls suggests that individuals with BD may have a distinct gut microbiota profile, with a greater abundance of *Clostridiaceae* and *Collinsella* (Mcintyre, Subramaniapillai, Shekotikhina, et al., 2019).

Together, these findings highlight the important role of bacteria in bidirectional communication with the brain and suggest that certain strains may, through novel mechanisms, prove to be useful therapeutic adjuncts in mental health. It is therefore important to understand how treatments that impact different pathways influence the gut microbiome. This area of investigation may provide new insights into the cause and treatment of mental illness. We therefore proposed to examine the longitudinal impact of infliximab, a drug that works by blocking the effects of tumor necrosis factor alpha (TNF‐α) on gut microbiome composition in individuals with BD.

## 2 METHODS

This study was conducted in accordance with the Canadian and international standards of Good Clinical Practice (International Conference on Harmonization guidelines for good clinical practice), applicable government regulations and had full research ethics approval from University Health Network Ethics Board (REB#14‐7369). We recruited men or women between 18 and 65 years of age who consented to be part of a randomized clinical trial examining the effects of infliximab, an antibody administered intravenously that is used for treating several chronic inflammatory diseases and works by blocking the effects of TNF‐α, on BD (Mcintyre, Subramaniapillai, Lee, et al., 2019). Eligible individuals were then subsequently asked to participate in the present microbiome substudy upon enrollment in the interventional clinical trial. Study personnel provided instructions and materials for stool sample collection to willing participants. All participants provided written, informed consent.

### 2.1 Stool sample collection and analysis

Stool samples were collected within 24 h preceding their first infliximab infusion (baseline), third infusion (week 6), and final study visit (week 12). Study participants were provided with stool collection kits, including ice packs and an insulated bag for transportation. Stool samples were frozen immediately upon receipt by study personnel and stored at −80°C for future analyses.

## 3 16S rRNA ANALYSIS

DNA extraction was carried out as previously described (Bharwani et al., 2016, 2020), including modifications to increase quantitative recovery of bacteria (Sibley et al., 2011, 2008; Whelan et al., 2014). 16S rRNA gene sequencing was carried out using a modified bar‐coded Illumina sequencing method (Bartram et al., 2011). Paired‐end reads of the V3 region were performed using the 341F and 518R primers (Muyzer et al., 1993), and 250 nt paired‐end sequencing was carried out on a MiSeq Illumina sequencer in the McMaster Genome Center. The data were processed through an in‐house bioinformatics pipeline. The reads were cleaned and trimmed of adapters using cutadapt and denoised to produce amplicon sequence variants (ASVs) using dada2. Taxonomy was assigned to the ASVs using the SILVA database version 1.38, after which all potential host sequences—those not assigned to Kingdoms Bacteria or Archaea, all reads with no Phylum assignment, and all reads assigned to Family Mitochondria—were removed. All reads with a mean abundance in the dataset of 10 or less were eliminated.

Alpha diversity was assessed using the Simpson and Shannon diversity indices. The principal component analysis was performed using Aitchison distances, and PERMANOVA was performed using the adonis function from the vegan package (v 2.5–7). To assess differential abundance of specific taxa, we used the package ANCOMBC, which models abundance using a generalized linear model framework while accounting for compositional and sampling effects. Data analysis was performed in R (v 4.0.3). The phyloseq (v 1.34), tidyverse (v 1.3), and ggplot2 (3.3.3) packages were used for data management and visualization, as well as in‐house R packages.

## 4 RESULTS

Seventeen participants (15 females) were recruited into the study (Table 1). The average age of the placebo and infliximab groups was 45.9 years (SD 10.7) and 46.13 years (SD 9.7), respectively. There were no significant differences in the baseline Montgomery‐Asberg Depression Rating Scale (MADRS) score (placebo, mean = 28.5, SD = 4.2; infliximab, mean = 34.71, SD = 6.8) and the baseline Clinical Global Impressions Scale (CGIS) score (placebo, mean = 4.38, SD = 0.5; infliximab, mean = 4.43, SD = 0.5). Two participants randomized to the infliximab group (one female, one male) were lost to follow‐up after randomization and not included in the final analysis. In parallel with the larger study from which this sample was taken (Lee et al., 2020), there was no significant between‐group differences of infliximab impact on MADRS score changes from baseline to endpoint.

**TABLE 1:** Demographic characteristics of the population

Participant characteristics			
	Infliximab (*n* = 9)	Placebo (*n* = 8)	*p*‐value
Age, years (mean, SD)	47.6 (10.3)	45.9(10.7)	.56
Sex, (*n*, female, %)	8 (88.9)	7 (87.5)	.73
Caucasian race (*n*, %)	2 (22.2)	3 (37.5)	.49
Baseline total score MADRS (mean, SD) CGIS (mean, SD)	32.7(7.6)	28.5(4.24)	.54
	4.22(.67)	4.4 (0.5)	.72

**FIGURE 1:**
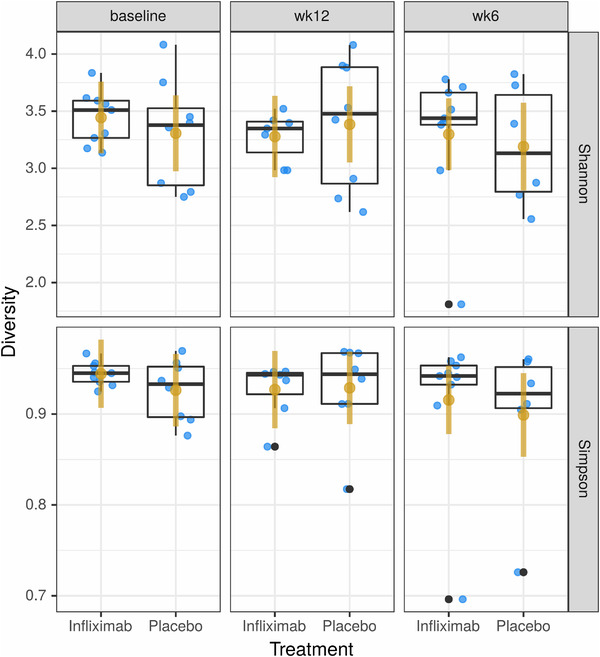
(a) Analysis of the Shannon Index revealed no differences in microbiota diversity between treatment groups. (b) Analysis of the Simpson Index revealed no differences in microbiota diversity between treatment groups

**FIGURE 2:**
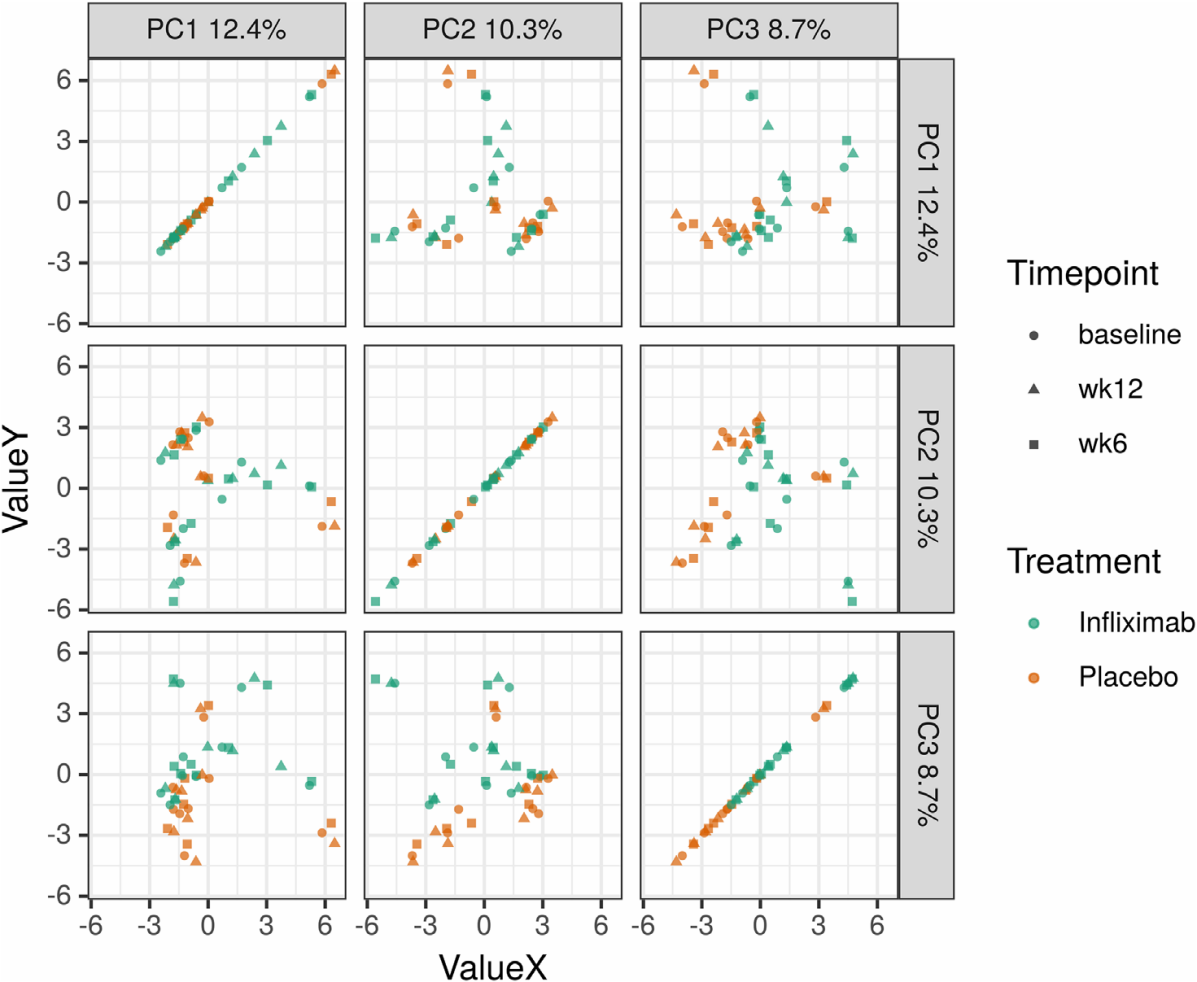
Aitchison distances revealed a lack of clustering of the microbiota by time point, but did reveal a small overall effect of treatment (Adonis; R^2^ = 0.053, *p *= .001) that was not significantly different at individual time points

To examine the effect of infliximab treatment on the microbiota, fecal samples were collected from patients at baseline prior to treatment initiation, and at 6 and 12 weeks following treatment. In order to ascertain the effects of infliximab on the diversity of the community, the Shannon and Simpson's Diversity indices were used to compare treatment across all time points. On the Shannon Index, there was no significant effect of either treatment or time on alpha‐diversity (Figure 1; *F*_5, 41 _= 0.268, *p *= .928). Similarly, there was no significant effect of either variable on Simpson's Diversity Index (Figure 1; *F*_5, 41 _= 0.539, *p *= 0.746). Next, in order to examine beta‐diversity and whether patient microbiomes clustered by treatment and time points, Aitchison distance was used to measure the dissimilarity among samples (Figure 2). Comparison of these distances revealed no clustering by time point (Adonis; *R* ^2^ = 0.011, *p *= 1), but did reveal a statistically significant, albeit small overall effect of treatment (Adonis; *R* ^2^ = 0.053, *p *= .001). Upon examination at individual time points however, there was no significant effect of treatment (week 6, *R* ^2^ = 0.06, *p *= .827; week 12, *R* ^2^ = 0.08, *p *= .143).

We then sought to examine whether specific microbial groups were differentially represented in certain samples. At both the ASV and genus level, there were no effects of either time or treatment on differential abundance.

## 5 DISCUSSION

Given that noninflammatory states are characterized by an intact gastrointestinal epithelial barrier, the mucosal immune system represents an important interface for microbe‐host signaling. The immune system responds to changes in lumen‐confined bacteria, while bacteria induce downstream changes in immune function, including in cytokine release and activation of immune cells (Collins et al., 2012; Murphy & Weaver, 2016). Additionally, there is a growing body of literature describing the role of peripheral immune signals in models of mental health conditions such as severe stress, post‐traumatic stress disorder, and MDD (Dantzer et al., 2008; Lindqvist et al., 2014; Sommershof et al., 2009). Although conventional mood stabilizers can alter the immune phenotype and down‐regulate the levels of inflammatory cytokines (Köhler et al., 2018; Pollmächer et al., 2000), and anti‐inflammatory agents have been described as having antidepressant properties (Akhondzadeh et al., 2009; Kappelmann et al., 2018; Nery et al., 2008), it is unclear whether these drugs impact the gut microbiota, either directly or indirectly, through alterations in the peripheral immune phenotype (Lee et al., 2020).

Upon examining the effect of infliximab on the microbiota, it was thus surprising to observe that 12 weeks of treatment had no effect on either the diversity of species (Figure 1) or the composition of the microbiota—either at the community level (Figure 2) or at the ASV or genus level—suggesting that systemic blockage of TNF alpha activity does not induce persistent changes in the microbiome. Interestingly, however, infliximab also did not significantly reduce depressive symptoms as compared with placebo in adults with bipolar depression, either in the primary infliximab trial or in our smaller cohort. In the lager trial, however, results from secondary analyses identified a subpopulation (i.e., those reporting physical and/or sexual abuse) that exhibited a significant reduction in depressive symptoms with infliximab treatment compared with placebo (Lee et al., 2020). This subpopulation has been associated with an increased proinflammatory response (Cohen‐Woods et al., 2018) and may speak to the role in infliximab in mood disorders linked to a heightened inflammatory phenotype. We were unable to investigate if this subpopulation had concomitant microbiome changes, however, given our small sample size. It is also worth noting that when there was no change in mood, there was, in parallel, no alteration in the gut microbiome. This is consistent with other work looking at the impact of pharmacotherapy on the gut microbiome in MDD, which found that while antidepressant medications alter the gut microbiota of patients with MDD, disparate effects are seen in responders versus nonresponders to treatment, supporting the concept of a microbiota phenotype associated with treatment response (Bharwani et al., 2020).

While it has previously been observed that infliximab infusion alters microbiota diversity, this was demonstrated in patients with inflammatory bowel diseases—a group of disorders characterized by aberrant local immune activity in the gut, along with altered intestinal microbiota structure and function (Akhondzadeh et al., 2009; Y. Wang et al., 2018). This may therefore account for the differences with the data described herein. A limitation of this study is the small sample size and lack of intermediary time point data. It is possible that any changes in the community were only apparent acutely and stabilized over the course of 12 weeks of treatment, which would parallel the efficacy data from the original Randmoized Controlled Trial (RCT) (Lee et al., 2020). Mapping symptom change with microbiome change is a valuable next step.

## 6 CONCLUSION

Our data indicate that no changes in the gut microbiota structure were detected after the use of infliximab in patients with BD who also did not benefit clinically from the treatment. Further clinical studies are necessary in order to determine whether infliximab may yet induce functional changes in the community that influence local and systemic immune activity. It does support the growing body of evidence that purports a change in the gut microbiome in those receiving pharmacotherapy when there is also a concomitant change in mood symptoms, which poses intriguing possibilities for the potential role of the microbiome in BD diagnosis and treatment.

## CONFLICT OF INTEREST

Dr. Roger McIntyre has received research grant support from Stanley Medial Research Institute, CIHR/GACD/Chinese Natural Research Foundation, and speaker/consultation fees from Lundbeck, Janssen, Shire, Purdue, Pfizer, Otsuka, Allergan, Takeda, Neurocrine, Sunovion, and Minerva. The other authors declare no conflict of interest.

### PEER REVIEW

The peer review history for this article is available at https://publons.com/publon/10.1002/brb3.2259.
